# Extended right surgical margin in distal pancreatectomy with celiac axis resection for pancreatic body cancer under the presence of replaced right hepatic artery; A case report

**DOI:** 10.1016/j.ijscr.2020.09.150

**Published:** 2020-09-24

**Authors:** Ryosuke Umino, Yuta Kobayashi, Masayuki Urabe, Satoshi Okubo, Junichi Shindoh, Masaji Hashimoto

**Affiliations:** a Hepatobiliary-pancreatic Surgery Division, Department of Gastroenterological Surgery, Toranomon Hospital, Tokyo, Japan; b Okinaka Memorial Institute for Medical Research, Tokyo, Japan

**Keywords:** BR, borderline resectable, CA, celiac axis, CHA, common hepatic artery, DP-CAR, distal pancreatectomy with celiac axis resection, FOLFIRINOX, 5-fluorouracil, leucovorin, irinotecan, and oxaliplatin, GDA, gastroduodenal artery, LGA, left gastric artery, NAC, neoadjuvant chemotherapy, PV, portal vein, PHA, proper hepatic artery, RHA, right hepatic artery, SMA, superior mesenteric artery, SMV, superior mesenteric vein, DP-CAR, Pancreatic body cancer, Replaced RHA

## Abstract

**Background:**

While distal pancreatectomy with celiac axis resection (DP-CAR) contributes to R0 resection for pancreatic body cancer, arterial blood flow to the liver from gastroduodenal artery is essential. However, in the presence of replaced right hepatic artery (r-RHA), extended DP-CAR (Ex-DP-CAR) in which the right edge of pancreatic resection includes the confluence of gastroduodenal artery (GDA) and proper hepatic artery (PHA) may be feasible. Herein, we report a patient with r-RHA and perform Ex-DP-CAR without reconstruction of PHA.

**Case presentation:**

A 39-year-old man with pancreatic cancer, cT4N0M0 (UICC 8th), underwent DP-CAR after neoadjuvant chemotherapy (NAC). After laparotomy, unlike the evaluation in preoperative imaging, the tumor was found to invade the confluence of GDA and PHA. After confirmation of arterial blood flow to the liver, GDA and PHA was ligated and Ex-DP-CAR was completed with R0 margin status. The postoperative course was uneventful, with no recurrence 18 months after the surgery.

**Conclusion:**

We performed Ex-DP-CAR with resection of the confluence of GDA and PHA in the presence of r-RHA, which has a potential role in expanding the surgical indications for R0 resection by reducing the risk of ischemic complications without reconstruction of arterial blood supply to the liver.

## Highlights

• We performed extended DP-CAR with resection of the confluence of GDA and PHA.
• The presence of r-RHA could reduce the risk of ischemic complications.
• Further, r-RHA could be a practical choice to expand surgical margin in DP-CAR.

## 1 Introduction

This case report is in line with the SCARE guidelines for surgical case reports [1]. Distal pancreatectomy with celiac axis resection (DP-CAR) is a surgical approach to secure a negative surgical margin (R0) in patients with pancreatic body cancer. Recent studies have reported that DP-CAR after neoadjuvant chemotherapy (NAC) may provide rather acceptable local control and contribute to R0 resection, resulting in the improvement of the survival outcomes in patients with borderline resectable (BR) pancreatic cancer [2], [3], [4], [5].

While DP-CAR may expand the resectability of pancreatic cancer, ischemic complications remain a significant concern with the sacrifice of the blood supply to the liver from common hepatic artery (CHA). Given the presence of an arterial arcade at the pancreatic head, blood flow to hepatobiliary system could be maintained as long as gastroduodenal artery (GDA) is preserved. In the patients with pancreatic body cancer involving the confluence of GDA and proper hepatic artery (PHA), resection might be given up or reconstruction of hepatic arterial flow should be performed in DP-CAR ensuring R0 resection. However, they are not suitable candidates for DP-CAR, because of an increased risk of severe complication and perioperative mortality in the excessive maneuver [6], [7].

Replaced right hepatic artery (r-RHA), outflowing from superior mesenteric artery (SMA) [8], is a frequently observed anatomic variation, representing 18% of the population [9]. Also, there are complex arterial communications around the bile duct in the hepatic hilus. Given arterial flow to the liver via r-RHA, the extent of the right sided pancreatic resection could be expanding with resection of the confluence of GDA and PHA.

Here we report extended DP-CAR (Ex-DP-CAR) with resection of the confluence of GDA and PHA, which might contribute to R0 resection for pancreatic body cancer in patients with r-RHA.

## 2 Case presentation

**Fig. 1:**
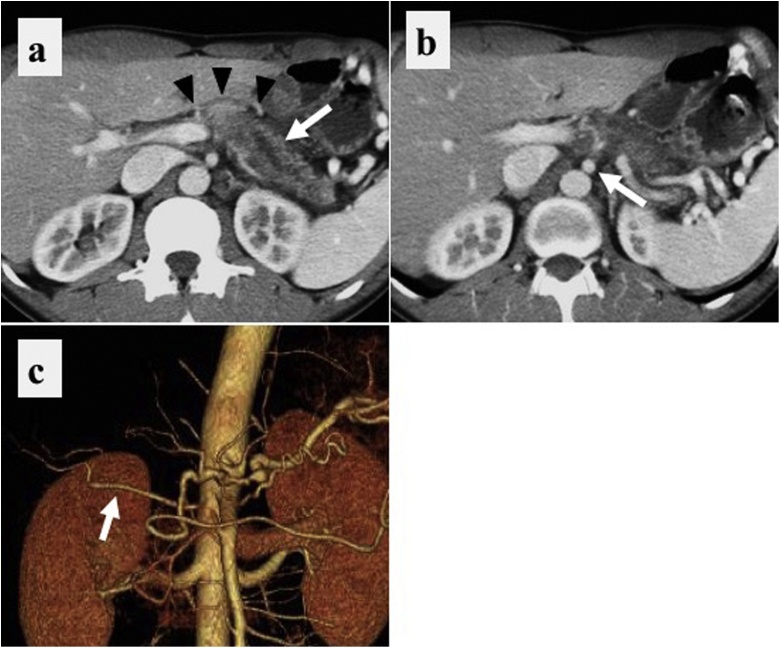
Preoperative computed tomography (Case #1) showed that. a: a hypodense lesion in the pancreatic body (black head) and dilation of the distal pancreatic duct (white head). b: the SMA free from the tumor (white arrow). c: the presence of the replaced RHA (white arrow).

A 39-year-old man presenting with a 5-month history of back and epigastric pain was referred to our institution with suspected pancreatic body cancer. Contrast-enhanced computed tomography (CT) showed a hypodense lesion in the pancreatic body with dilation of the distal pancreatic duct. The tumor infiltrated the celiac artery (CA) without involvement of GDA (Fig. 1a, b). CT also confirmed the presence of r-RHA (Fig. 1c). The portal vein and superior mesenteric vein were free from the tumor invasion. There were no enlarged lymph nodes or any metastatic lesions.

With the diagnosis of BR pancreatic cancer (T4N0M0), the patient received 5 cycles of FOLFIRINOX (5-fluorouracil, leucovorin, irinotecan, and oxaliplatin), and after that, underwent surgery.

**Fig. 2:**
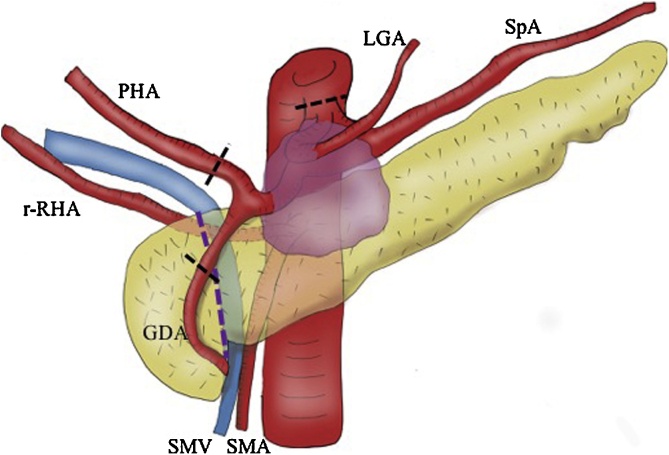
Operative findings and procedure. The tumor was found to involve the root of the GDA and the PHA. After confirming complementary arterial flow to the left hemiliver from the replaced RHA through the hepatic hilum, the PHA and the GDA were ligated.

**Fig. 3:**
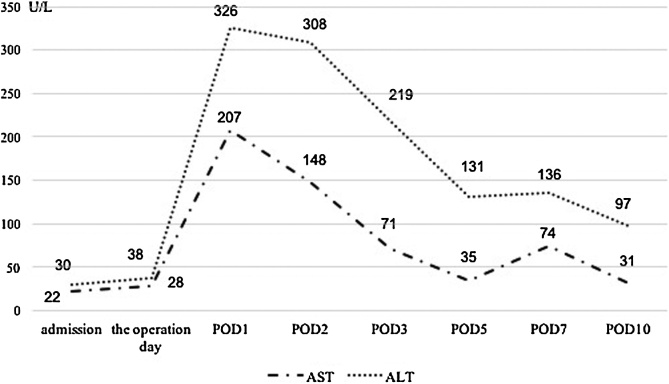
Postoperative transaminases. The postoperative course was uneventful with no evidence of ischemic complications.

At laparotomy, inconsistent with the evaluation of preoperative imaging, the tumor was found to involve the confluence of GDA and PHA. After clamp for PHA, doppler ultrasonography confirmed arterial flow in the liver. Therefore, PHA and GDA were ligated and the pancreas was divided with a surgical stapling device (ECHELON 60 mm, Gold cartridge) along with the right edge of the portal vein. Finally, we cut the left gastric artery (LGA) and CA, and finished Ex- DP-CAR (Fig. 2). The postoperative course was uneventful, with no ischemic complications, and the patient was discharged 10 days after the surgery (Fig. 3).

Histopathological examination of the resected surgical specimen revealed a moderately differentiated invasive ductal adenocarcinoma of the pancreas (T3), with no direct invasion of the wall of CA or GDA. The surgical margin was negative for cancer cells. There was no evidence of lymph node involvement and the pathological stage was ⅡA according to the UICC 8th classification [10]. Postoperative chemotherapy with S-1 (120 mg/body) was carried out for 6 months and the patient remains well with no evidence of recurrence nor ischemic complication such as liver abscess and gastric ischemia at 18 months after the surgery.

## 3 Discussion

The prognosis of pancreatic cancer is poor [11]. Surgical resection is only curative option and margin negative resection (R0) is essential to cure the pancreatic cancer. Combined resection of the invaded artery is one of the significant points of R0 resection of the cancer. With the recent developments in surgical techniques and chemo-radiotherapy, surgical outcomes have also improved through expansion of the surgical indications for BR pancreatic cancer [12], [13].

DP-CAR is a surgical procedure for the pancreas body cancer which invades CA or proximal part of CHA with an incidence of high mortality and morbidity [14]. One of the limitations of this DP-CAR is the extent of invasion along with CHA because the confluence of GDA and PHA cannot be resected for the preserving hepatic arterial flow from SMA. However, in the presence of r-RHA and complementary arterial blood supply to the liver, we considered that Ex-DP-CAR with resection of the confluence of GDA and PHA was successfully completed in this case, without reconstruction of arterial blood flow to the liver.

Theoretically, ischemia to the hepatobiliary system and the pancreas after DP-CAR can be avoided with arterial flow from SMA via the arcade at the pancreatic head to GDA. However, resection of major arteries is associated with an increased risk of liver abscess and/or ischemic gastropathy and thereby, of the perioperative mortality [15]. Although the arterial clamp is routinely performed during such invasive procedures that deprive the liver of its major arterial supply, arterial insufficiency could result even after careful preservation of the arcade at the pancreatic head. To reduce the risk of ischemic complications, preoperative coil embolization of CHA or arterial bypass using the middle colic artery or splenic artery has been reported [16], [17]. However, when the confluence of GDA or PHA is involved, arterial reconstruction is needed to maintain the arterial blood flow to the liver. Nevertheless, according to a recent meta-analysis, arterial reconstruction is associated with an increased risk of perioperative mortality and a 50% decrease in the 1-year survival rate as compared to that in patients undergoing pancreatectomy without any vascular reconstructions [14]. As such, the indication for surgery should be carefully discussed in advanced cases. In our case, the tumor was found to have extended to the confluence of GDA and PHA. We considered that detachment of the tumor from surrounding vessels should not be performed for R0 resection. Therefore, we performed Ex-DP-CAR without preserving pancreatic artery arcade.

The noteworthy finding in our case, based on our experience reported herein, is that the presence of r-RHA might be beneficial in such a situation and allow arterial flow to the liver to be maintained even after deprivation of supply from PHA. According to Michel’s classification of anatomic variations of the hepatic artery, r-RHA is present in 18% of the population [9]. Usually, r-RHA arises from SMA, travelling behind or through the head of the pancreatic parenchyma. Previous authors reported that r-RHA should be carefully identified to prevent injury or to achieve a negative surgical margin during pancreaticoduodenectomy [18], [19], [20]. Although r-RHA is not touched during the procedure of distal pancreatectomy and its clinical role has not been reported, the present outcomes suggest a potential role of r-RHA in expanding the surgical indications for R0 resection in DP-CAR by reducing the risk of ischemic complications without reconstruction of arterial blood supply to the liver.

## 4 Conclusion

We performed Ex-DP-CAR with resection of the confluence of GDA and PHA under the presence of r-RHA. Our case is the first report that the flow from r-RHA is effectively utilized in DP-CAR. Although the surgical indications should be considered carefully in patients with advanced pancreatic cancer, the outcomes in our cases reported here may warrant careful expansion of the surgical indications for pancreatic body cancer in the era of multidisciplinary treatment.

## Declaration of Competing Interest

The authors report no declarations of interest.

## Funding

No funding was obtained from the private or public sector for this research.

## Ethical approval

All procedures followed were performed in accordance with the ethical standards laid down in the 1964 Declaration of Helsinki and its later amendments.

## Consent

A statement of consent was included in the end of the manuscript.

## Author contribution

All authors contributed to the study conception and design. The first draft of the manuscript was written by R. Umino. J. Shindoh revised the manuscript. M. Hashimoto is the chairperson of our department and supervised the writing of the manuscript. All authors commented on previous versions and approved the final manuscript.

## Guarantor

Masaji Hashimoto, a manager of Hepatobiliary-pancreatic Surgery Division, Department of Gastroenterological Surgery, Toranomon Hospital.

## Provenance and peer review

Not commissioned, externally peer-reviewed.

## Availability of data and materials

The datasets used and/or analysed during the current study are available from the corresponding author on reasonable request.
